# Epilepsy mortality in Wales during COVID-19

**DOI:** 10.1016/j.seizure.2021.11.017

**Published:** 2022-01

**Authors:** Helen Daniels, Arron S Lacey, David Mikadze, Ashley Akbari, Beata Fonferko-Shadrach, Joe Hollinghurst, Ronan A Lyons, Mark I Rees, Inder MS Sawhney, Robert H Powell, Michael P Kerr, W Owen Pickrell

**Affiliations:** a Swansea University Medical School, Swansea University, Swansea, SA2 8PP, United Kingdom; b Morriston Hospital, Swansea Bay University Health Board, Swansea, Wales, United Kingdom; c Faculty of Medicine and Health, University of Sydney, Australia; d Institute of Psychological Medicine and Clinical Neurosciences, Cardiff University School of Medicine, Cardiff, Wales, United Kingdom

**Keywords:** Data linkage, Electronic health records, Pandemic, COVID-19

## Abstract

**Purpose:**

The COVID-19 pandemic has increased mortality worldwide and those with chronic conditions may have been disproportionally affected. However, it is unknown whether the pandemic has changed mortality rates for people with epilepsy. We aimed to compare mortality rates in people with epilepsy in Wales during the pandemic with pre-pandemic rates.

**Methods:**

We performed a retrospective study using individual-level linked population-scale anonymised electronic health records. We identified deaths in people with epilepsy (DPWE), i.e. those with a diagnosis of epilepsy, and deaths associated with epilepsy (DAE), where epilepsy was recorded as a cause of death on death certificates. We compared death rates in 2020 with average rates in 2015–2019 using Poisson models to calculate death rate ratios.

**Results:**

There were 188 DAE and 628 DPWE in Wales in 2020 (death rates: 7.7/100,000/year and 25.7/100,000/year). The average rates for DAE and DPWE from 2015 to 2019 were 5.8/100,000/year and 23.8/100,000/year, respectively. Death rate ratios (2020 compared to 2015–2019) for DAE were 1.34 (95%CI 1.14–1.57, *p*<0.001) and for DPWE were 1.08 (0.99–1.17, *p* = 0.09). The death rate ratios for non-COVID deaths (deaths without COVID mentioned on death certificates) for DAE were 1.17 (0.99–1.39, *p* = 0.06) and for DPWE were 0.96 (0.87–1.05, *p* = 0.37).

**Conclusions:**

The significant increase in DAE in Wales during 2020 could be explained by the direct effect of COVID-19 infection. Non-COVID-19 deaths have not increased significantly but further work is needed to assess the longer-term impact.

## 1 Introduction

Coronavirus disease 2019 (COVID-19) has had a devastating effect on people's health and has resulted in increased mortality worldwide [1]. Those with chronic conditions, such as epilepsy, may be disproportionally affected and more susceptible to COVID-19 hospitalisation and death [2].

In addition to the direct effects of COVID-19, the pandemic has affected people with epilepsy in several ways. These include the impact of COVID-19 on mental health, health services, and healthcare seeking behaviour [3,4]. Non-pharmaceutical interventions to decrease virus transmission may have increased psychological stress, disrupted sleep, and reduced contact with vital social support networks [3]. There has been a significant decrease in consultations with health services for non-COVID-19 illnesses, including epilepsy, particularly at the onset of the pandemic. Specialist epilepsy services in Wales adapted rapidly to restrictions imposed during the pandemic, where virtual (telephone and video) epilepsy clinics became the norm from March 2020 onwards. This very significant restriction on face-to-face contact in specialist and primary care settings will have altered patient experience [4,5]. Each of these factors can potentially increase seizure frequency and, subsequently, the risk of death in people with epilepsy.

People with epilepsy already have an increased mortality risk when compared to the general population [6]. The timely assessment of changes in mortality is therefore important to target resources and potentially save lives. To our knowledge, there have been no population level studies on mortality rates in people with epilepsy during the COVID-19 pandemic. We used individual-level, population-scale routinely collected, electronic health record (EHR) data to compare mortality rates in people with epilepsy during the COVID-19 pandemic with background mortality rates.

## 2 Method

We used the Secure Anonymised Information Linkage (SAIL) Databank, which contains anonymised individual-level, population-scale routinely collected EHR data sources from multiple different sources. These include hospital admission and demographic data for the complete Welsh population (3.1 million) and primary care records for 80% of the population [7,8].

Our study population consisted of all individuals living in Wales registered with a Welsh General Practice (GP) contributing data to SAIL during 1/1/2015–31/12/2020. We used primary care, death certificate and demographic data and analysed epilepsy-related deaths in two ways:

***Deaths associated with epilepsy (DAE*)** had an International Classification of Diseases version 10 (ICD10) code for epilepsy in any of the ten cause of death positions on death certificate data (see Supplementary information S1 for codes) [9]; this included people who did not necessarily receive a diagnosis of epilepsy before death.

***Deaths in people with epilepsy (DPWE)*** were deaths from all causes in individuals with a *diagnosis of epilepsy* before death. Using a previously validated method, we defined a person as having epilepsy if their GP record contained Read codes for an epilepsy diagnosis (S2) and a prescription of an anti-seizure medication (S3) on two separate occasions within 6 months [10]. These include people of all ages with epilepsy.

We calculated monthly and annual death rates by dividing DAE and DPWE by the total study population on January 1st of the relevant study year. COVID-19 deaths were defined as deaths with the ICD-10 codes U07.1 and U07.2 recorded in any position on death certificates. The first COVID-19 death in Wales was recorded in March 2020.

We used Poisson regression models to calculate death rate ratios for each group in: (i) 2020 compared with 2015–2019 and (ii) each month in 2020 compared the same month in 2015–2019. Data from each individual year (2015–2020) and month were used in the model with the study population as an offset. R version 3.5.2 was used for analyses.

This study was approved by the SAIL independent Information Governance Review Panel (IGRP) (ref 0696). The Research Ethics Service has confirmed that SAIL projects using anonymised, routinely collected data do not require research ethics committee approval.

## 3 Results

**Fig. 1:**
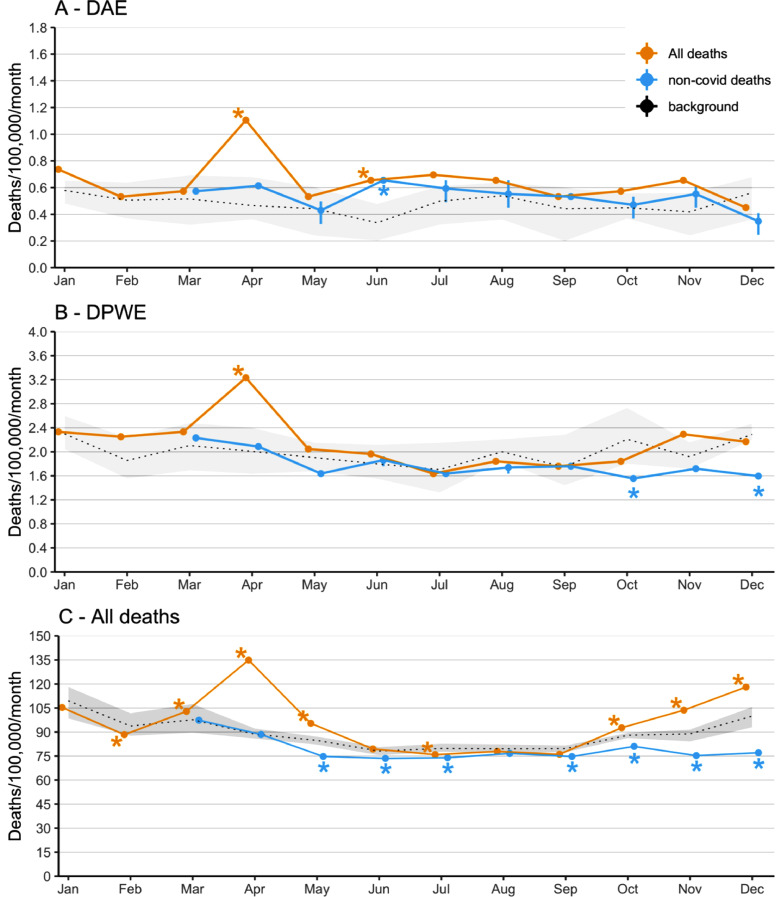
All and non-COVID death rates per month in 2020 for: (A) deaths associated with epilepsy (DAE), (B) deaths in people with epilepsy (DPWE) and (C) all deaths in Wales. Note there are different scales on the vertical axis. The grey shading indicates the range of the corresponding monthly death rates for the years 2015–2019. The dotted black line represents mean death rates for 2015–2019. Orange points and lines are total deaths, blue points and lines are non-COVID-19 deaths (deaths without COVID-19 on the death certificate). *Asterisks represent death rates that are significantly different to the 2015–2019 background rate. A range of values (blue error bars) has been displayed for some non-COVID-19 death rates as exact values cannot be displayed due to small numbers of COVID-19 deaths (<5).

We analysed 14.9 million patient-years of data. The mean number of people with epilepsy was 24,560 and the mean population was 2481,516 for each study year (2015–2020) (mean epilepsy prevalence=0.99%). During the study period, there were 904 deaths associated with epilepsy (DAE) and 3595 deaths in people with epilepsy (DPWE). See Table 1 for deaths per year and Fig. 1 and Supplementary Table 4 for deaths per month.

**Table 1:** Deaths associated with epilepsy (DAE) and in people with epilepsy (DPWE) in 2020 and preceding years. All deaths and non-COVID deaths are shown in separate rows. Death rates are per 100,000 per year. Death rate ratios are the ratio of 2020 death rates to 2015–2019 rates with confidence intervals from Poisson regression models. Death rate ratios that are significantly different to background rates are shown in bold. Non-COVID-19 deaths are deaths without COVID-19 mentioned on the death certificate. § DAE and DPWE populations are the total number of people registered with a Welsh GP that contribute data to SAIL (covering approx. 80% of the Welsh population). The population for all Welsh deaths is the total Welsh population. Populations are estimated from figures taken on 1st January.

	2015–2019			2020			Death rate ratio
	Number of deaths (mean per year)	Population^§^ (mean per year)	Death rate/100,000/year (mean)	Number of deaths	Population^§^	Death rate/100,000/year	
DAE DAE (non-Covid) DPWE DPWE (non-Covid)	143 143 593 593	2489,114	5.75 5.75 23.84 23.84	188 165 628 548	2443,623	7.69 6.75 25.70 22.43	**1.34 (1.14–1.57, *p*<0.001)** 1.17 (0.99–1.39, *p* = 0.06) 1.08 (0.99–1.17, *p* = 0.09) 0.96 (0.87–1.05, *p* = 0.37)
All Welsh deaths All Welsh deaths (non-Covid)	33,388 33,388	3125,876	1068 1068	36,279 31,105	3152,900	1151 986.60	**1.08 (1.07–1.09, *p*<0.001)** **0.92 (0.91–0.94, *p*<0.001)**

DAE in 2020 increased significantly when compared to DAE in 2015–2019 [death rate ratio=1.34 (95% CI 1.14–1.57,*p*<0.001)];12% were COVID-related. Non-COVID DAE in 2020 had a smaller, non-statistically significant increase [death rate ratio=1.17 (0.99–1.39,*p* = 0.06)].

There was a non-significant increase in DPWE in 2020 when compared to 2015–2019, [death rate ratio=1.08 (0.99–1.17,*p* = 0.09)];13% were COVID-related. Non-COVID DPWE did not change significantly when compared to DPWE in 2015–2019 (death rate ratio=0.96 (0.87–1.05, *p* = 0.37)].

## 4 Discussion

There was a significant increase in deaths associated with epilepsy (DAE) in Wales in 2020 compared with DAE 2015–2019. There was a smaller, non-statistically significant, increase in deaths in people with epilepsy (DPWE).

Most of this excess mortality can be explained by deaths directly caused by COVID-19 given that non-COVID-19 DAE and DPWE have not increased. This means that the indirect effects of the pandemic, such as disruptions to healthcare, are unlikely to have increased deaths in both groups. It may be that changes during the pandemic such as more access to remote consultations, reduced rates of other infections, and more family/carers available at home have not unduly disadvantaged people with epilepsy.

The trends in DAE and DPWE broadly reflect the pattern for all deaths in Wales. Monthly patterns in DAE and DPWE (Fig. 1, S4) also reflect monthly patterns in all-cause mortality with a significant peak occurring in April 2020 during the peak of the “first wave” of COVID-19 in Wales. There were lower rates in DAE and DPWE during summer months when COVID-19 rates were low.

Background rates of DPWE are around four times higher than DAE (23.8/100,000/year compared with 5.8/100,000/year). DAE are more likely to be directly related to epilepsy prompting the recording of the diagnosis on the death certificate, for example, deaths due to sudden death in epilepsy (SUDEP), status epilepticus, or injuries directly related to seizures. DPWE likely include more deaths not directly related to epilepsy.

We have been able to rapidly analyse contemporaneous individual-level population-scale healthcare data. We have used a validated method of ascertaining people with an *established diagnosis of epilepsy* to study deaths in people with epilepsy as well as for people with *deaths associated with epilepsy*. We have also completed a detailed comparison with background mortality rates in 2015–2019. Our analysis will allow for further, more detailed, assessment of the impact of the second UK wave of Covid-19 infections on people living with epilepsy and the longer-term impact on mortality.

Epilepsy is a heterogeneous disorder with many different causes, affecting people of all ages, with a high proportion of comorbidities. We have not accounted for these differences. It may be that particular subgroups, for example older people with epilepsy or people with epilepsy and comorbid intellectual disability or dementia, have had differing mortality rates when compared with people with epilepsy as a whole, particularly given the strong association between age and COVID-19 mortality. Our method for identifying people with epilepsy would have included a proportion who have been seizure free at time of death, and this may have biased results. People living in areas of increased socioeconomic deprivation have increased epilepsy prevalence and have been disproportionally affected by COVID—we have not accounted for this in our analysis [11]. We also did not account for the position that epilepsy was recorded on the death certificate, which may influence the strength of association of epilepsy with the death.

There is likely a delay in epilepsy diagnoses recording in primary care and we were not able to record new diagnosis of epilepsy made from October 2020. This means that DPWE may be slightly higher than recorded here although these factors will not have affected our figures for DAE. Sudden and unexpected death in epilepsy (SUDEP) is a rare but devastating cause of death in people with epilepsy. We have not specifically looked at SUDEP as it is poorly coded in routinely collected data [12].

## Conclusion

In this population-scale study of epilepsy mortality we have found that deaths associated with epilepsy increased significantly in 2020 when compared with rates from 2015 to 2019. This is largely explained by COVID-19 related deaths as non-COVID-19 deaths associated with epilepsy and non-COVID-19 deaths in people with epilepsy have not increased significantly. Further research is necessary, such as sub-group analysis by age, epilepsy severity, and co-morbidity, to assess the longer-term impact of COVID on epilepsy, especially in vulnerable groups.

## Declaration of Competing Interest

None of the authors have any conflicts of interest to disclose.

## Appendix Supplementary materials

Image, application 1
