# Supplementary material for: Epilepsy mortality in Wales during COVID-19
Source: Seizure. 2022 Jan;94:39–42. doi: 10.1016/j.seizure.2021.11.017 (PMC8626872; doi:10.1016/j.seizure.2021.11.017)
Supplement: Supplementary file 1 [file mmc1.docx]

# Supplementary Information

### S1 - List of ICD10 codes used to for identifying epilepsy as a cause of death

### S2 - List of Read codes used to signify a diagnosis of epilepsy

### S3 - List of antiepileptic drugs and their read codes

### S4 - Table of deaths associated with epilepsy and in people with epilepsy in 2020 by month

### S1 - List of ICD10 codes used for identifying epilepsy as a cause of death

G40.0 Localization-related (focal)(partial) idiopathic epilepsy and epileptic syndromes with seizures of localized onset

G40.1 Localization-related (focal)(partial) idiopathic epilepsy and epileptic syndromes with seizures of localized onset

G40.2 Localization-related (focal)(partial) symptomatic epilepsy and epileptic syndromes with complex partial seizures

G40.3 Generalized idiopathic epilepsy and epileptic syndromes

G40.4 Other generalized epilepsy and epileptic syndromes

G40.5 Special epileptic syndromes

G40.6 Grand mal seizures, unspecified (with or without petit mal)

G40.7 Petit mal, unspecified, without grand mal seizures

G40.8 Other epilepsy

G40.9 Epilepsy, unspecified

G41.0 Grand mal status epilepticus

G41.1 Petit mal status epilepticus

G41.2 Complex partial status epilepticus

G41.8 Other status epilepticus

G41.9 Status epilepticus, unspecified

R56.8 Other and unspecified convulsions

### S2 – List of Read codes used to signify a diagnosis of epilepsy

F25B. Alcohol-induced epilepsy

F25y4 Benign Rolandic epilepsy

F2545 Complex partial epileptic seizure

F25y3 Complex partial status epilepticus

F25y0 Cursive (running) epilepsy

F25C. Drug-induced epilepsy

F259. Early infant epileptic encephalopathy wth suppression bursts

F25.. Epilepsy

1O30. Epilepsy confirmed

F25z. Epilepsy NOS

F2544 Epileptic automatism

F2503 Epileptic seizures - akinetic

F2502 Epileptic seizures - atonic

F2512 Epileptic seizures - clonic

F2513 Epileptic seizures - myoclonic

F2514 Epileptic seizures - tonic

F25y1 Gelastic epilepsy

F251. Generalised convulsive epilepsy

F251z Generalised convulsive epilepsy NOS

F250. Generalised nonconvulsive epilepsy

F250z Generalised nonconvulsive epilepsy NOS

F2510 Grand mal (major) epilepsy

F2516 Grand mal seizure

F253. Grand mal status

F2560 Hypsarrhythmia

F256. Infantile spasms

F256z Infantile spasms NOS

F2550 Jacksonian, focal or motor epilepsy

F2504 Juvenile absence epilepsy

F25A. Juvenile myoclonic epilepsy

F257. Kojevnikov's epilepsy

F2505 Lennox-Gastaut syndrome

F2543 Limbic system epilepsy

F25y2 Locl-rlt(foc)(part)idiop epilep&epilptic syn seiz locl onset

F25D. Menstrual epilepsy

F2511 Neonatal myoclonic epilepsy

667B. Nocturnal epilepsy

F25y. Other forms of epilepsy

F25yz Other forms of epilepsy NOS

F251y Other specified generalised convulsive epilepsy

F250y Other specified generalised nonconvulsive epilepsy

F25y5 Panayiotopoulos syndrome

F254. Partial epilepsy with impairment of consciousness

F254z Partial epilepsy with impairment of consciousness NOS

F255. Partial epilepsy without impairment of consciousness

F255z Partial epilepsy without impairment of consciousness NOS

F255y Partial epilepsy without impairment of consciousness OS

F2500 Petit mal (minor) epilepsy

F252. Petit mal status

F25F. Photosensitive epilepsy

F258. Post-ictal state

F2541 Psychomotor epilepsy

F2542 Psychosensory epilepsy

F2501 Pykno-epilepsy

F2561 Salaam attacks

F2551 Sensory induced epilepsy

F2556 Simple partial epileptic seizure

F2552 Somatosensory epilepsy

F25X. Status epilepticus, unspecified

F25E. Stress-induced epilepsy

F2540 Temporal lobe epilepsy

F2515 Tonic-clonic epilepsy

SC200 Traumatic epilepsy

F2555 Unilateral epilepsy

F2553 Visceral reflex epilepsy

F2554 Visual reflex epilepsy

### S3 – List of anti-epileptic drugs and their Read codes used to identify cases of epilepsy

dn3e.*ARBIL MR 200mg m/r tablets

dn3f. *ARBIL MR 400mg m/r tablets

dn2.. *BECLAMIDE

dn2z. *BECLAMIDE 500mg tablets

dnc1. *CLOBAZAM SLS 10mg capsules

do1z. *DIAZEPAM 20mg/4mL injection

do1B. *DIAZEPAM 20mg/5mL RecTubes

dn53. *EMESIDE 250mg capsules

dn3A. *EPIMAZ 100mg tablets

dn3B. *EPIMAZ 200mg tablets

dn3C. *EPIMAZ 400mg tablets

dn51. *ETHOSUXIMIDE 250mg capsules

dn52. *ETHOSUXIMIDE 250mg/5mL elixir

dn5y. *ETHOSUXIMIDE 250mg/5mL elixir

dn79. *GARDENAL 200mg/1mL injection

dn7a. *LUMINAL 15mg tablets

dn7b. *LUMINAL 30mg tablets

dn7c. *LUMINAL 60mg tablets

dn21. *NYDRANE 500mg tablets

dnba. *ORLEPT 200mg e/c tablets

dnbb. *ORLEPT 500mg e/c tablets

do52. *PARALDEHYDE injection 10mL

do51. *PARALDEHYDE injection 5mL

dn98. *PENTRAN 100mg tablets

dn97. *PENTRAN 50mg tablets

dn63. *PROMINAL 200mg tablets

dn61. *PROMINAL 30mg tablets

dn62. *PROMINAL 60mg tablets

do13. *STESOLID 20mg/4mL injection

dn3H. *TERIL CR 200mg m/r tablets

dn3I. *TERIL CR 400mg m/r tablets

dn55. *ZARONTIN 250mg capsules

dn1y. ACETAZOLAMIDE [EP] 250mg tablets

dn1z. ACETAZOLAMIDE [EP] 500mg injection

dn1x. ACETAZOLAMIDE [EP] 500mg m/r capsules

dn1.. ACETAZOLAMIDE [EPILEPSY]

do41. ATIVAN [EP] 4mg/mL injection

dn3J. CARBAGEN SR 200mg m/r tablets

dn3K. CARBAGEN SR 400mg m/r tablets

dn3.. CARBAMAZEPINE

dn3y. CARBAMAZEPINE 100mg chewable tablets

dn31. CARBAMAZEPINE 100mg tablets

dn3z. CARBAMAZEPINE 100mg/5mL sugar free liquid

dn3v. CARBAMAZEPINE 125mg suppositories

dn3x. CARBAMAZEPINE 200mg chewable tablets

dn3a. CARBAMAZEPINE 200mg m/r tabs

dn32. CARBAMAZEPINE 200mg tablets

dn3w. CARBAMAZEPINE 250mg suppositories

dn3b. CARBAMAZEPINE 400mg m/r tabs

dn33. CARBAMAZEPINE 400mg tablets

dnc.. CLOBAZAM [EPILEPSY ONLY]

do3.. CLOMETHIAZOLE EDISYLATE [CENTRAL NERVOUS SYSTEM USE]

do3z. CLOMETHIAZOLE EDISYLATE 8mg/mL intravenous infusion

dn4.. CLONAZEPAM [EPILEPSY CONTROL]

do2.. CLONAZEPAM [STATUS EPILEPSY]

dn4w. CLONAZEPAM 0.5mg/5mL sugar free oral solution

do2z. CLONAZEPAM 1mg/1mL injection

dn4z. CLONAZEPAM 2mg tablets

dn4x. CLONAZEPAM 2mg/5mL sugar free oral solution

dn4y. CLONAZEPAM 500microgram tablets

dn... CONTROL OF EPILEPSY

dnh1. CONVULEX 150mg e/c capsules

dnh2. CONVULEX 300mg e/c capsules

dnh3. CONVULEX 500mg e/c capsules

dnh7. DEPAKOTE 250mg e/c tablets

dnh8. DEPAKOTE 500mg e/c tablets

dns1. DIACOMIT 250mg capsules

dns3. DIACOMIT 250mg/sachet powder for oral suspension

dns2. DIACOMIT 500mg capsules

dns4. DIACOMIT 500mg/sachet powder for oral suspension

dn12. DIAMOX [EP] 250mg tablets

dn13. DIAMOX [EP] 500mg injection

dn11. DIAMOX [EP] 500mg m/r capsules

do11. DIAZEMULS [EP] 10mg/2mL injection

do1.. DIAZEPAM [EPILEPSY USE]

do1y. DIAZEPAM 10mg/2.5mL rectal solution

do19. DIAZEPAM 10mg/2.5mL RecTubes

do1v. DIAZEPAM 10mg/2mL emulsion injection

do1w. DIAZEPAM 10mg/2mL injection

do1t. DIAZEPAM 2.5mg/1.25mL rectal solution

do1A. DIAZEPAM 2.5mg/1.25mL RecTubes

do1u. DIAZEPAM 20mg/5mL rectal solution

do1x. DIAZEPAM 5mg/2.5mL rectal solution

do18. DIAZEPAM 5mg/2.5mL RecTubes

dn54. EMESIDE 250mg/5mL syrup

do61. EPANUTIN [EP] 250mg/5mL injection

dn95. EPANUTIN 100mg capsules

dn93. EPANUTIN 25mg capsules

dn96. EPANUTIN 300mg capsules

dn81. EPANUTIN 30mg/5mL suspension

dn94. EPANUTIN 50mg capsules

dn82. EPANUTIN 50mg Infatabs

dnb1. EPILIM 100mg crushable tablets

dnb2. EPILIM 200mg e/c tablets

dnb4. EPILIM 200mg/5mL sugar free liquid

dnb5. EPILIM 200mg/5mL syrup

dnb3. EPILIM 500mg e/c tablets

dnbc. EPILIM CHRONO 200 m/r tablets

dnbd. EPILIM CHRONO 300 m/r tablets

dnbe. EPILIM CHRONO 500 m/r tablets

dnbQ. EPILIM CHRONOSPHERE 100mg/sachet m/r granules

dnbU. EPILIM CHRONOSPHERE 1g/sachet m/r granules

dnbR. EPILIM CHRONOSPHERE 250mg/sachet m/r granules

dnbS. EPILIM CHRONOSPHERE 500mg/sachet m/r granules

dnbP. EPILIM CHRONOSPHERE 50mg/sachet m/r granules

dnbT. EPILIM CHRONOSPHERE 750mg/sachet m/r granules

dnb6. EPILIM IV 400mg/4mL injection

dnbF. EPISENTA 150mg m/r capsules

dnbO. EPISENTA 1g/10mL solution for injection

dnbI. EPISENTA 1g/sachet m/r granules

dnbG. EPISENTA 300mg m/r capsules

dnbD. EPISENTA 300mg/3mL solution for injection

dnbH. EPISENTA 500mg/sachet m/r granules

dnbB. EPIVAL CR 300mg m/r tablets

dnbC. EPIVAL CR 500mg m/r tablets

dnu.. ESLICARBAZEPINE

dnu2. ESLICARBAZEPINE ACETATE 800mg tablets

dn5.. ETHOSUXIMIDE

dn5x. ETHOSUXIMIDE 250mg capsules

dn5z. ETHOSUXIMIDE 250mg/5mL syrup

dni.. FOSPHENYTOIN SODIUM

dni1. FOSPHENYTOIN SODIUM 750mg/10mL injection concentrate

dnj.. GABAPENTIN

dnj1. GABAPENTIN 100mg capsules

dnj2. GABAPENTIN 300mg capsules

dnjx. GABAPENTIN 300mg capsules/600mg tablets titration pack

dnj3. GABAPENTIN 400mg capsules

dnjy. GABAPENTIN 600mg tablets

dnjz. GABAPENTIN 800mg tablets

dnl5. GABITRIL 10mg tablets

dnl6. GABITRIL 15mg tablets

dnl4. GABITRIL 5mg tablets

do31. HEMINEVRIN [CNS] 8mg/mL intravenous infusion

dnr1. INOVELON 100mg tablets

dnr2. INOVELON 200mg tablets

dnr3. INOVELON 400mg tablets

dno5. KEPPRA 100mg/mL s/f oral solution

dno3. KEPPRA 1g tablets

dno1. KEPPRA 250mg tablets

dno2. KEPPRA 500mg tablets

dno6. KEPPRA 500mg/5mL solution for injection

dno4. KEPPRA 750mg tablets

dnt.. LACOSAMIDE

dntA. LACOSAMIDE 100mg tablets

dntB. LACOSAMIDE 150mg tablets

dnt8. LACOSAMIDE 15mg/1mL sugar free liquid

dntC. LACOSAMIDE 200mg tablets

dnt7. LACOSAMIDE 200mg/20mL solution for injection

dnt9. LACOSAMIDE 50mg tablets

dnf9. LAMICTAL 100mg dispersible tablets

dnf4. LAMICTAL 100mg tablets

dnfD. LAMICTAL 200mg tablets

dnf8. LAMICTAL 25mg dispersible tablets

dnf6. LAMICTAL 25mg tablets

dnfJ. LAMICTAL 2mg dispersible tablets

dnf3. LAMICTAL 50mg tablets

dnf7. LAMICTAL 5mg dispersible tablets

dnfF. LAMICTAL MONOTHERAPY 25mg starter pack

dnfH. LAMICTAL NON-VALPROATE ADD-ON 50mg starter pack

dnfG. LAMICTAL VALPROATE ADD-ON 25mg starter pack

dnf.. LAMOTRIGINE

dnfC. LAMOTRIGINE 100mg dispersible tablets

dnf2. LAMOTRIGINE 100mg tablets

dnfE. LAMOTRIGINE 200mg tablets

dnfB. LAMOTRIGINE 25mg dispersible tablets

dnf5. LAMOTRIGINE 25mg tablets

dnfz. LAMOTRIGINE 2mg dispersible tablets

dnf1. LAMOTRIGINE 50mg tablets

dnfA. LAMOTRIGINE 5mg dispersible tablets

dno.. LEVETIRACETAM

dnov. LEVETIRACETAM 100mg/mL s/f oral solution

dnox. LEVETIRACETAM 1g tablets

dnoz. LEVETIRACETAM 250mg tablets

dnoy. LEVETIRACETAM 500mg tablets

dnou. LEVETIRACETAM 500mg/5mL solution for injection

dnow. LEVETIRACETAM 750mg tablets

do4.. LORAZEPAM [EPILEPSY]

dnp4. LYRICA 100mg capsules

dnp5. LYRICA 150mg capsules

dnp6. LYRICA 200mg capsules

dnp8. LYRICA 225mg capsules

dnp1. LYRICA 25mg capsules

dnp7. LYRICA 300mg capsules

dnp2. LYRICA 50mg capsules

dnp3. LYRICA 75mg capsules

dn6.. METHYLPHENOBARBITAL

dn6z. METHYLPHENOBARBITONE 200mg tablets

dn6x. METHYLPHENOBARBITONE 30mg tablets

dn6y. METHYLPHENOBARBITONE 60mg tablets

dna1. MYSOLINE 250mg tablets

dna2. MYSOLINE 250mg/5mL oral suspension

dna3. MYSOLINE 50mg tablets

dnj4. NEURONTIN 100mg capsules

dnj5. NEURONTIN 300mg capsules

dnj9. NEURONTIN 300mg capsules/600mg tablets titration pack

dnj6. NEURONTIN 400mg capsules

dnj7. NEURONTIN 600mg tablets

dnj8. NEURONTIN 800mg tablets

dng2. NOOTROPIL 1.2g tablets

dng3. NOOTROPIL 33% oral solution

dng1. NOOTROPIL 800mg tablets

dnbA. ORLEPT 200mg/5mL sugar free liquid

dnb9. ORLEPT STARTER PACK 200mg e/c tablets x10

dnm.. OXCARBAZEPINE

dnmx. OXCARBAZEPINE 150mg tablets

dnmy. OXCARBAZEPINE 300mg tablets

dnmz. OXCARBAZEPINE 600mg tablets

dnmw. OXCARBAZEPINE 60mg/mL sugar free oral suspension

do5.. PARALDEHYDE

dn7.. PHENOBARBITAL

dn74. PHENOBARBITAL 100mg tablets

dn71. PHENOBARBITAL 15mg tablets

dn7d. PHENOBARBITAL 15mg/5mL elixir

dn78. PHENOBARBITAL 200mg/1mL injection

dn72. PHENOBARBITAL 30mg tablets

dn73. PHENOBARBITAL 60mg tablets

dn77. PHENOBARBITONE 15mg/10mL elixir

dn75. PHENOBARBITONE SODIUM 30mg tablets

dn76. PHENOBARBITONE SODIUM 60mg tablets

dn8.. PHENYTOIN

dn92. PHENYTOIN 100mg tablets

dn8y. PHENYTOIN 30mg/5mL suspension

dn83. PHENYTOIN 50mg chewable tablets

dn91. PHENYTOIN 50mg tablets

dn8z. PHENYTOIN 90mg/5mL sugar free suspension

dn9.. PHENYTOIN SODIUM

do6.. PHENYTOIN SODIUM [STATUS EPILEPSY]

dn9z. PHENYTOIN SODIUM 100mg capsules

do6z. PHENYTOIN SODIUM 250mg/5mL injection

dn9x. PHENYTOIN SODIUM 25mg caps

dn9w. PHENYTOIN SODIUM 300mg capsules

dn9y. PHENYTOIN SODIUM 50mg capsules

dng.. PIRACETAM

dng5. PIRACETAM 1.2g tablets

dng6. PIRACETAM 333.3mg/mL oral solution

dng4. PIRACETAM 800mg tablets

dnp.. PREGABALIN

dnpv. PREGABALIN 100mg capsules

dnpw. PREGABALIN 150mg capsules

dnpu. PREGABALIN 200mg capsules

dnps. PREGABALIN 225mg capsules

dnpz. PREGABALIN 25mg capsules

dnpt. PREGABALIN 300mg capsules

dnpy. PREGABALIN 50mg capsules

dnpx. PREGABALIN 75mg capsules

dna.. PRIMIDONE

dnay. PRIMIDONE 250mg tablets

dnaz. PRIMIDONE 250mg/5mL oral suspension

dnax. PRIMIDONE 50mg tablets

dni2. PRO-EPANUTIN 750mg/10mL injection concentrate

dnv.. RETIGABINE

dnv8. RETIGABINE 100mg tablets

dnv9. RETIGABINE 200mg tablets

dnvA. RETIGABINE 300mg tablets

dnvB. RETIGABINE 400mg tablets

dnv7. RETIGABINE 50mg tablets

dnvC. RETIGABINE 50mg+100mg tablets initiation pack

do21. RIVOTRIL 1mg/1mL injection

dn42. RIVOTRIL 2mg tablets

dn41. RIVOTRIL 500micrograms tablets

dnr.. RUFINAMIDE

dnrz. RUFINAMIDE 100mg tablets

dnry. RUFINAMIDE 200mg tablets

dnrx. RUFINAMIDE 400mg tablets

dne4. SABRIL 500mg powder sachets

dne2. SABRIL 500mg tablets

dnb.. SODIUM VALPROATE

dnbv. SODIUM VALPROATE 100mg crushable tablets

dnbo. SODIUM VALPROATE 100mg/sachet m/r granules

dnbJ. SODIUM VALPROATE 150mg m/r capsules

dnbN. SODIUM VALPROATE 1g/10mL solution for injection

dnbM. SODIUM VALPROATE 1g/sachet m/r granules

dnbw. SODIUM VALPROATE 200mg crushable tablets

dnb7. SODIUM VALPROATE 200mg e/c tablets

dnbr. SODIUM VALPROATE 200mg m/r tablets

dnby. SODIUM VALPROATE 200mg/5mL sugar free liquid

dnbz. SODIUM VALPROATE 200mg/5mL syrup

dnbp. SODIUM VALPROATE 250mg/sachet m/r granules

dnbK. SODIUM VALPROATE 300mg m/r capsules

dnbs. SODIUM VALPROATE 300mg m/r tablets

dnbE. SODIUM VALPROATE 300mg/3mL solution for injection

dnbu. SODIUM VALPROATE 400mg/4mL injection

dnb8. SODIUM VALPROATE 500mg e/c tablets

dnbt. SODIUM VALPROATE 500mg m/r tablets

dnbx. SODIUM VALPROATE 500mg tablets

dnbL. SODIUM VALPROATE 500mg/sachet m/r granules

dnbn. SODIUM VALPROATE 50mg/sachet m/r granules

dnbq. SODIUM VALPROATE 750mg/sachet m/r granules

do... STATUS EPILEPTICUS DRUGS

do12. STESOLID [EP] 10mg/2mL injection

do15. STESOLID 10mg/2.5mL rectal solution

do14. STESOLID 5mg/2.5mL rectal solution

dnsw. STIRIPENDOL 500mg/sachet powder for oral suspension

dns.. STIRIPENTOL

dnsz. STIRIPENTOL 250mg capsules

dnsx. STIRIPENTOL 250mg/sachet powder for oral suspension

dnsy. STIRIPENTOL 500mg capsules

dn3c. TEGRETOL 100mg chewable tablets

dn34. TEGRETOL 100mg tablets

dn37. TEGRETOL 100mg/5mL sugar free liquid

dn3D. TEGRETOL 125mg suppositories

dn3d. TEGRETOL 200mg chewable tablets

dn35. TEGRETOL 200mg tablets

dn3E. TEGRETOL 250mg suppositories

dn36. TEGRETOL 400mg tablets

dn38. TEGRETOL RETARD 200mg m/r tabs

dn39. TEGRETOL RETARD 400mg m/r tabs

dnl.. TIAGABINE

dnl2. TIAGABINE 10mg tablets

dnl3. TIAGABINE 15mg tablets

dnl1. TIAGABINE 5mg tablets

dn3F. TIMONIL RETARD 200mg m/r tablets

dn3G. TIMONIL RETARD 400mg m/r tablets

dnk5. TOPAMAX 100mg tablets

dnk6. TOPAMAX 200mg tablets

dnk8. TOPAMAX 25mg tablets

dnk4. TOPAMAX 50mg tablets

dnkB. TOPAMAX SPRINKLE 15mg capsules

dnkC. TOPAMAX SPRINKLE 25mg capsules

dnkE. TOPAMAX SPRINKLE 50mg capsules

dnk.. TOPIRAMATE

dnk2. TOPIRAMATE 100mg tablets

dnk9. TOPIRAMATE 15mg beads in capsules

dnk3. TOPIRAMATE 200mg tablets

dnkA. TOPIRAMATE 25mg beads in capsules

dnk7. TOPIRAMATE 25mg tablets

dnkD. TOPIRAMATE 50mg beads in capsules

dnk1. TOPIRAMATE 50mg tablets

dnm1. TRILEPTAL 150 tablets

dnm2. TRILEPTAL 300 tablets

dnm3. TRILEPTAL 600 tablets

dnm4. TRILEPTAL 60mg/mL sugar free oral suspension

dnv2. TROBALT 100mg tablets

dnv3. TROBALT 200mg tablets

dnv4. TROBALT 300mg tablets

dnv5. TROBALT 400mg tablets

dnv1. TROBALT 50mg tablets

dnv6. TROBALT tablets initiation pack

do16. VALIUM [EP] 10mg/2mL injection

do17. VALIUM [EP] 20mg/4mL injection

dnh.. VALPROIC ACID

dnh4. VALPROIC ACID 150mg e/c capsules

dnhz. VALPROIC ACID 250mg e/c tablets

dnh5. VALPROIC ACID 300mg e/c capsules

dnh6. VALPROIC ACID 500mg e/c capsules

dnhy. VALPROIC ACID 500mg e/c tablets

dne.. VIGABATRIN

dne3. VIGABATRIN 500mg powder sachets

dne1. VIGABATRIN 500mg tablets

dnt4. VIMPAT 100mg tablets

dnt5. VIMPAT 150mg tablets

dnt2. VIMPAT 15mg/1mL sugar free liquid

dnt6. VIMPAT 200mg tablets

dnt1. VIMPAT 200mg/20mL solution for injection

dnt3. VIMPAT 50mg tablets

dn56. ZARONTIN 250mg/5mL syrup

dnu1. ZEBINIX 800mg tablets

dnq6. ZONEGRAN 100mg capsules

dnq4. ZONEGRAN 25mg capsules

dnq5. ZONEGRAN 50mg capsules

dnq.. ZONISAMIDE

dnq3. ZONISAMIDE 100mg capsules

dnq1. ZONISAMIDE 25mg capsules

dnq2. ZONISAMIDE 50mg capsule

### S4 – Table of deaths associated with epilepsy and in people with epilepsy in 2020 by month

| **Deaths associated with epilepsy** | | | | | | | |  |
| --- | --- | --- | --- | --- | --- | --- | --- | --- |
| **Month** | **2015–2019 rate** | **2020 rate** | **2020 (non-COVID) rate** | | **Death rate ratio** | **Death rate ratio (non-COVID)** | |  |
| Jan | 0.579 | 0.737 | - | 1.27 (0.74–2.08) | | | - | |
| Feb | 0.506 | 0.532 | - | 1.05 (0.55–1.84) | | | - | |
| Mar | 0.515 | 0.573 | 0.573 | 1.11 (0.60–1.93) | | | 1.11 (0.60–1.93) | |
| Apr | 0.466 | 1.110 | 0.610 | **2.37 (1.48–3.70)** | | | 1.32 (0.72–2.26) | |
| May | 0.442 | 0.532 | 0.491–0.327* | 1.20 (0.63–2.13) | | | * | |
| Jun | 0.337 | 0.655 | 0.655 | **1.94 (1.06–3.38)** | | | **1.94 (1.06–3.38)** | |
| Jul | 0.498 | 0.696 | 0.491–0.655* | 1.40 (0.79–2.33) | | | * | |
| Aug | 0.539 | 0.655 | 0.450–0.614* | 1.22 (0.68–2.04) | | | * | |
| Sep | 0.442 | 0.532 | 0.530 | 1.20 (0.63–2.13) | | | 1.20 (0.63–2.13) | |
| Oct | 0.450 | 0.573 | 0.368–0.532* | 1.27 (0.68–2.22) | | | * | |
| Nov | 0.418 | 0.655 | 0.450–0.614* | 1.57 (0.87–2.68) | | | * | |
| Dec | 0.562 | 0.455 | 0.246–0.409* | 0.80 (0.40–1.45) | | | ***** | |
| **Deaths in people with epilepsy** | | | | | | | |  |
| **Month** | **2015–2019 rate** | **2020 rate** | **2020 (non-COVID) rate** | **Death Rate ratio** | | | **Death rate ratio (non-COVID)** | |
| Jan | 2.297 | 2.333 | - | 1.01 (0.76–1.34) | | | - | |
| Feb | 1.856 | 2.251 | - | 1.21 (0.90–1.61) | | | - | |
| Mar | 2.105 | 2.333 | 2.169–2.292* | 1.11 (0.82–1.46) | | | * | |
| Apr | 2.007 | 3.233 | 2.090 | **1.61 (1.24–2.06)** | | | 1.04 (0.76–1.39) | |
| May | 1.903 | 2.046 | 1.640 | 1.07 (0.78–1.44) | | | 0.86 (0.61–1.19) | |
| Jun | 1.799 | 1.964 | 1.760–1.923* | 1.09 (0.79–1.48) | | | * | |
| Jul | 1.704 | 1.637 | 1.430 | 0.96 (0.68–1.33) | | | 0.96 (0.68–1.33) | |
| Aug | 2.001 | 1.842 | 1.637–1.800* | 0.92 (0.66–1.25) | | | * | |
| Sep | 1.746 | 1.760 | 1.560 | 1.01 (0.72–1.38) | | | 1.01 (0.72–1.38) | |
| Oct | 2.209 | 1.842 | 1.560 | 0.83 (0.60–1.13) | | | **0.70 (0.49–0.98)** | |
| Nov | 1.919 | 2.292 | 1.720 | 1.19 (0.88–1.58) | | | 0.90 (0.64–1.23) | |
| Dec | 2.289 | 2.169 | 1.600 | 0.95 (0.70–1.26) | | | **0.70 (0.49–0.96)** | |
| **All deaths in Wales** | | | | | | | |  |
| **Month** | **2015–2019 rate** | **2020 rate** | **2020 (non-COVID) rate** | **Death Rate ratio** | | | **Death rate ratio (non-COVID)** | |
| Jan | 109.52 | 105.33 | - | 0.96 (0.93–1.00) | | | - | |
| Feb | 93.75 | 88.36 | - | **0.94 (0.90–0.98)** | | | - | |
| Mar | 97.65 | 102.89 | 97.37 | **1.05 (1.01–1.09)** | | | 1.00 (0.96–1.04) | |
| Apr | 88.71 | 134.92 | 88.49 | **1.52 (1.47–1.57)** | | | 1.00 (0.96–1.04) | |
| May | 84.76 | 95.44 | 74.76 | **1.13 (1.08­–1.17)** | | | **0.88 (0.84–0.92)** | |
| Jun | 78.13 | 79.42 | 73.52 | 1.02 (0.97–1.06) | | | **0.94 (0.90–0.98)** | |
| Jul | 79.74 | 75.90 | 73.93 | **0.95 (0.91–0.99)** | | | **0.93 (0.89–0.97)** | |
| Aug | 79.54 | 77.90 | 76.66 | 0.98 (0.94–1.02) | | | 0.96 (0.92–1.01) | |
| Sep | 79.66 | 76.09 | 74.72 | 0.96 (0.91–1.00) | | | **0.94 (0.90–0.98)** | |
| Oct | 88.02 | 92.68 | 81.04 | **1.05 (1.01–1.10)** | | | **0.92 (0.88–0.96)** | |
| Nov | 88.86 | 106.65 | 75.30 | **1.17 (1.12–1.21)** | | | **0.85 (0.81–0.88)** | |
| Dec | 99.93 | 118.08 | 77.07 | **1.18 (1.14–1.22)** | | | **0.77 (0.74–0.80)** | |

**Deaths associated with epilepsy and in people with epilepsy in 2020 by month**. Death rates are per 100,000 per month. Death rate ratios are the monthly ratios of 2020 death rates to 2015–2019 rates with confidence intervals from Poisson regression models. Death rate ratios that are significantly different to background rates are shown in bold. Non-covid deaths are deaths without COVID-19 mentioned on the death certificate and are shown from March 2020 onwards (the first COVID death was reported in March 2020 in Wales). *exact numbers cannot be shown due to small (<5) numbers of COVID deaths in these groups
